# Low-Power Voltage Transformer Smart Frequency Modeling and Output Prediction up to 2.5 kHz, Using Sinc-Response Approach

**DOI:** 10.3390/s20174889

**Published:** 2020-08-29

**Authors:** Abbas Ghaderi, Alessandro Mingotti, Lorenzo Peretto, Roberto Tinarelli

**Affiliations:** Department of Electrical, Electronic and Information Engineering–Guglielmo Marconi, Alma Mater Studiorum–University of Bologna, Viale del Risorgimento 2, 40136 Bologna, Italy; abbas.ghaderi2@unibo.it (A.G.); lorenzo.peretto@unibo.it (L.P.); roberto.tinarelli3@unibo.it (R.T.)

**Keywords:** sinc-response, single-frequency test, distorted waveform test, ratio error, phase displacement, transfer function, voltage transformers

## Abstract

The instrument transformers scenario is moving towards the adoption of a new generation of low-power instrument transformers. This disruptive change also requires that the modeling, characterization, and testing of those devices must be improved. Therefore, this study focuses on a smart approach developed by the authors in a previous study to estimate the output of low-power voltage transformers (LPVT). The approach—which is based on a sort of modeling in the frequency domain (the so-called sinc-response)—allows obtaining the behavior of the LPVT at rated and distorted conditions. Experimental tests performed on off-the-shelf devices confirm the applicability and effectiveness of the proposed approach when estimating the output response of LPVTs.

## 1. Introduction

In smart grid paradigms, knowledge of power network state variables a key requirement for the power-system stability. To measure the node voltages and the branch currents as state variables, instrument transformers (ITs) are deployed. Because of the widespread use of distributed generation (DG) especially at the medium voltage (MV) level, studies on MV ITs are hot topics nowadays. Inductive-voltage transformers (VTs) and inductive current transformers (CTs) form the legacy or conventional ITs family, while the low-power voltage transformers (LPVTs) and low-power current transformers (LPCTs) form the new generation of low-power instrument transformers (LPITs). LPVTs feature a smaller size and lighter weight as compared to conventional Its, which make them distinguished in smart grids considering the DGs. Moreover, LPITs have a wider frequency band which makes them suitable for power-quality measurements, as well as other applications that involves a substantial harmonic content and frequencies different from the typical 50–60 Hz range.

ITs are mainly used for protection and measurement purposes. Protection ITs need to detect rapidly changing voltages or currents, while measurement ITs need to measure voltage and current with as much accuracy as possible. Measurement errors may lead to wrong estimates of state variables during the state-estimation (SE) process and load-flow measurements in MV network; therefore, resulting in network instability whether or not wrong corrective actions are implemented according to the wrong measurements obtained. The measured power may also be used for billing purposes, which means that it is mandatory for the ITs to be compliant with the limits fixed by their accuracy class (AC). For the above reasons, studies on the modeling, characterization, uncertainty analysis, and accuracy assessment under different influence quantities are some of the most studied topics in this area.

For example, [1,2] introduce a test bed for LPITs for industrial applications and methods for their testing. For accuracy assessment, ratio error and phase displacement are the two main parameters to be measured. In terms of accuracy assessment, Ref. [3,4,5] represent the efforts on the installation, traceability and challenges of integration in LPVTs. The uncertainty analysis of the ITs and their calibration test bed as a key factor for measurement, is performed in [6,7,8,9,10], while the nonlinearity compensation is assessed in [11] for voltage and current ITs. The application of ITs in the power network considering different network features is analyzed in research works such as [12,13,14,15]. The standard IEC 61869-6 referred in [16] generalizes the requirements for LPITs.

The modeling of ITs at first glance looks already familiar due to the fundamental operation of ITs, but it requires new techniques to be developed. Such techniques are required because the measurement ITs model must be very accurate in order to be reliable for electric utilities and to provide useful information for the network management. In other words, the research works on modeling are closely linked to those on the ITs accuracy. Ref. [17,18] are some of the research works for LPIT modeling, while [19] is an attempt to inductive CT modeling by accurate measurement of the model parameters. In [20], what obtained in [19] is used for prediction of the ratio error and phase displacement under controlled influence quantities which can be used for online calibration of ITs.

The impulse-response (IR) test is a well-known method for dynamic systems modeling. In this work, the authors start from the IR test concept for modeling a passive LPVT rated for a MV network. There is a large limit on IR test performance on LPVTs. LPVTs based on capacitive dividers are only slightly dependent on frequency. Normally, this dependency is neglected in industrial applications, while it is critical when LPVTs need to be accurately characterized for different frequencies even though the accuracy may slightly change. In the literature, IR tests and sweep frequency (known as frequency-response) tests are performed for different applications. Ref. [21,22,23,24,25,26] are some research works on IR test performed on mechanical and electrical systems. For frequency-response tests, Ref. [27,28,29] are the research works done on VTs. Ref. [30] represents another frequency characterization of VTs, while [31] analyzes the frequency characterization of VT under network interruptions. For LPCTs, [32] introduces a new Rogowski coil design to measure a wide pulse current. Regardless the ITs, IR and frequency-response tests have a long history on power transformers. Ref. [33] is one example of IR tests and frequency-response test for power-transformer diagnostics and [34] represents IR test for power-transformer dielectric design.

To overcome these accuracy IR measurement limitations, this work applies a new technique using a sinc signal for the characterization of LPVTs. This approach is called sinc-response (SR) test in correlation with IR test terminology. In contrast to impulse signal, a sinc function has the advantage of being easily measurable by conventional acquisition systems. However, in the SR test, the maximum frequency is limited to a certain range to be set during the design of the sinc signal (as detailed in the core of the work). The limited frequency is not an issue for the purpose of the work, which is to characterize the passive LPVT up to power quality frequency range (2.5 kHz) (see [16]).

The SR approach a signal was introduced in [35] and applied for the prediction of the Rogowski coil output, but without focusing on ratio error and phase-displacement aspects.

In this work, an electric equivalent circuit of the LPVT under test is introduced in Section 2, while the concept of SR and IR testing is discussed in Section 3. Section 4 deals with the theoretical and mathematical design of the sinc signal. The adopted experimental setup is introduced in Section 5. In Section 6, the required experimental test procedures, model extraction using SR test, and simulations for output prediction are discussed. The simulation and experimental test results are reported and discussed in Section 7, while in Section 8, we summarize the main achievements of the work and present the main conclusions.

## 2. LPVT Model

In this work, the considered LPVT is a capacitive one. The reason for this choice is that such kind of LPVT is increasing adopted when an LPVT is considered for installation in the distribution network. However, any kind of LPVT may be tested with the proposed approach.

**Figure 1:**
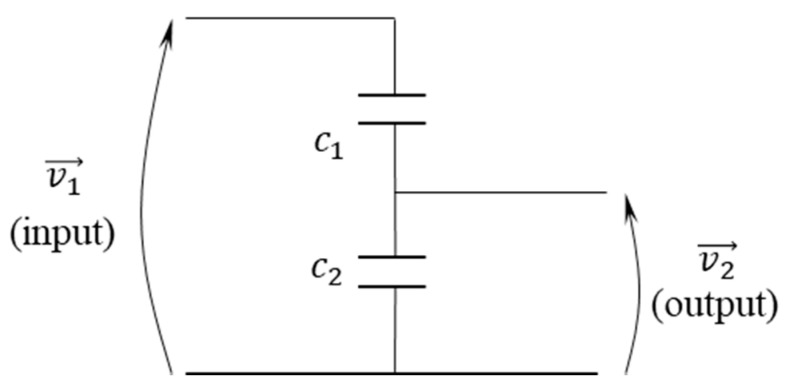
Low-power voltage transformers (LPVT) electric equivalent circuit.

LPVTs based on capacitive dividers are mainly designed in active and passive configurations. The difference between the two categories concerns the design limitation and the customer preference. Figure 1 shows an electric equivalent circuit of a passive LPVTs based on a pure capacitive divider. Normally, the secondary capacitor is an embedded one, while the primary capacitor is a built-in capacitor obtained from the chassis of the device. For this reason, the built-in primary capacitance may vary from device to device, hence leading to different transformation ratios for different products. To normalize the transformation ratio and phase displacement of each product to the nominal values—after the characterization process—correction factors are provided to the customer by the manufacturer. It should be noted that in any case the accuracy class is not compromised.

On the other hand, in active LPVTs an active electronic amplifier is added to the passive LPVTs to bring the transformation ratio and phase displacement to the nominal ones so that all final products keep the nominal values declared on the nameplate without introducing extra correction factors.

In case of considering an ideal LPVT (composed of ideal capacitors) showed in Figure 1, the transformation ratio expressed in Equation (1) is simply a gain with zero phase displacement and it is not frequency-dependent.

(1) $$v_{2}=\frac{c_{1}+c_{2}}{c_{2}}v_{1}$$

Equation (1), represents the input–output relation of the ideal LPVT where $v_{1}$ and $v_{2}$ are the primary and secondary voltages, respectively, while $c_{1}$ and $c_{2}$ are the primary and secondary capacitors, respectively.

The ideal LPVT cannot be obtained in practical situations and the non-idealities are mainly due to two reasons: (a) resistive or conduction leakage in the dielectric material of capacitors and (b) phase displacement between real and imaginary part of dielectric permeability in capacitors. Both mentioned properties are slightly variable with frequency, which lets the overall ratio error and phase displacement to be slightly frequency-dependent. The word “slightly” in the term “slightly frequency dependency” is used to emphasis the fact that, in contrast to Equation (1), the transfer function of an LPVT is not simply a gain. Therefore, the LPVTs can be considered dynamic systems which accurate modeling by using SR test builds the core of the present research.

## 3. IR Test Method vs. SR Test Method

### 3.1. IR Test Method

The characterization of a dynamic or static system using IR test, corresponds to the system input–output relation in different frequencies. The range of the frequency and the frequency component selection depends on the closeness of the impulse signal to the ideal one. According to Equation (2), an ideal impulse signal implementation is a crucial task to perform. The challenge of implementing such signal mainly concerns the width of the signal around t = 0. The importance of the implementation of a quasi-ideal impulse signal is even greater if the system under test is not highly frequency-dependent.

(2) $$\delta \left(t\right)=0\;for\;t\neq 0,\;\int_{-\infty }^{+\infty }\delta \left(t\right)\;dt=1$$

In Equation (2) δ(t) indicates the Dirac delta distribution function. In case of a slightly frequency-dependent system, the impulse-response is highly similar to the input impulse signal. The slightly difference between input impulse signal and impulse response represents the system model included in the transfer function. In this case, highly accurate signal acquisition techniques are required for both input and output signals to establish the input–output relation. In particular, high sampling rate for the analog-to-digital converter (ADC) is required for the transfer function extraction. In practice, even high-resolution data-acquisition boards (DAQ) and oscilloscopes are not capable of input impulse signal and impulse-response signal acquisition for some slightly frequency-dependent systems such as LPVTs. Consequently, the input–output relation is not well established and the procedure for model extraction fails.

### 3.2. SR Test Method

**Figure 2:**
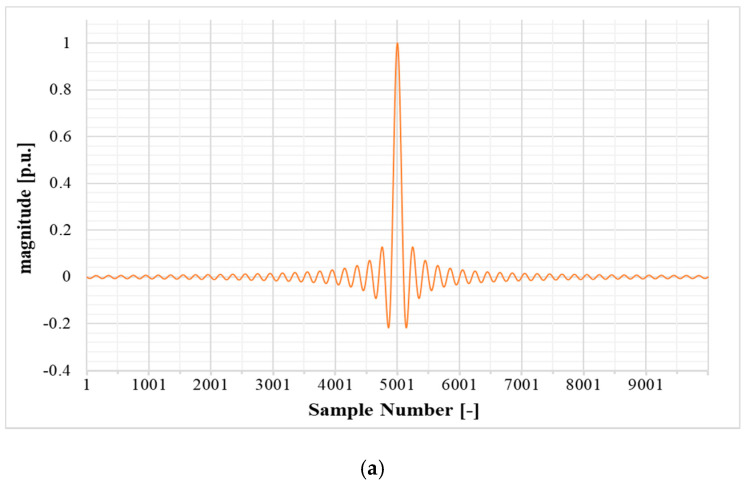

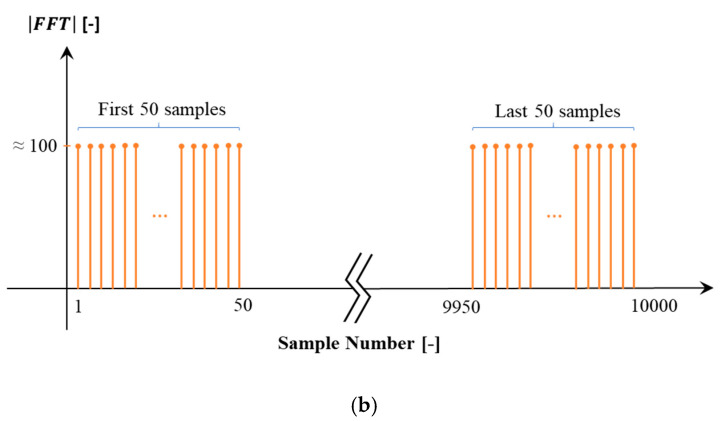
Designed sinc signal. (**a**) Time domain; (**b**) discrete Fourier transform (DFT) magnitude.

The sinc-response test represents the characterization of the system using the sinc signal instead of an impulse signal. The sinc function defined as:

(3) $$Sinc\left(x\right)=\frac{sin\left(x\right)}{x}$$

has a frequency domain characteristic similar to that of an impulse signal, but in a limited range and with a frequency step defined during the design of the sinc signal. The ideal impulse signal in frequency domain represents a constant function with unity value ranging from −∞ to +∞ while, a sampled sinc signal represents a rectangular window. Figure 2 shows the designed sinc signal for this work and the corresponding discrete Fourier transform (DFT) using 49 side lobes and N = 10,000 samples.

According to the sinc signal repetition frequency and the considered number of lobes, the width of each lobe is wide enough to be sampled using conventional ADCs and to be measured using conventional DAQs and oscilloscopes. This feature is the key to overcome the challenge of accurate measurement of input and output signals in IR test by using SR test instead of it. This is the approach adopted in this work.

On the other hand, the disadvantage of SR test is the limitation on the frequency range that the LPVT is characterized for. However, the 2.5 kHz power quality frequency range (adopted in this work) allows a proper testing of the LPVTs, guaranteeing the desired accuracy and resolution for the characterization process.

Of course, even if omitted in Figure 2, the signal has a direct component which is completely neglectable, compared to the main signal and out of scope of this work.

Summarizing, the three main characteristics of a sinc signal are: (i) number of lobes; (ii) number of samples which represents the magnitude of each frequency component in frequency domain and (iii) repetition frequency of the sinc signal itself. The relation between the mentioned three features and the procedure for the discrete sinc signal design is expressed in Section 4.

## 4. Sinc-Signal Design

The goal is to specify the frequency corresponding to any sample, within the rectangular window in the DFT graph of Figure 2, to design the sinc signal to operate in the power quality range (2.5 kHz) and to calculate the Fourier series coefficient for each frequency component. In this work, the sinc function is designed and sampled to be generated, amplified and applied to the LPVT under test. According to Figure 2, the design variables to be considered are: the DFT amplitude, frequency range and frequency resolution (frequency step). In the sinc function design procedure, a tradeoff between the three variables should be obtained.

### 4.1. Sinc Function Fourier-Transform Analysis

Both time domain and frequency domain sinc signals are sampled data; this is why DFT is used. It is recalled that the discrete time Fourier transform (DTFT) applied to a sampled signal, outputs the continuous frequency domain signal with period of 2π as:

(4) $$X\left(e^{j\omega }\right)=\overset{+\infty }{\underset{n=-\infty }{\sum }}x\left(n\right)e^{-j\omega n}$$

In order to extract the DFT, the periodic function DTFT is sampled in frequency domain with respect to the variable *ω*. One important consideration is that both time and frequency domain sampling frequency is equal to the same value N. Applying DTFT sampling, (4) turns into:

(5) $$X\left(k\right)=\overset{+\infty }{\underset{n=-\infty }{\sum }}x\left(n\right)e^{-j\left(\frac{2\pi }{N}k\right)n}$$

As Figure 2 shows, the width of the window is represented in terms of number of Samples N. It is well known that the width of the window represents the range of the frequency components contained in the designed sinc signal. In order to specify the frequency corresponding to the number of samples in the width of the window, it is useful to define the following variables:

- $f_{o}$ [Hz]: the sinc signal repetition frequency in time domain;
- T_o_ [s]: the period of the sinc signal (1/$f_{o}$);
- $f_{s}$ [Sa/s]: sampling frequency of the DAQ;
- $T_{s}$ [s]: the sampling time (1/$f_{s}$);
- N: the number of samples in only one period of the sinc signal.

**Figure 3:**
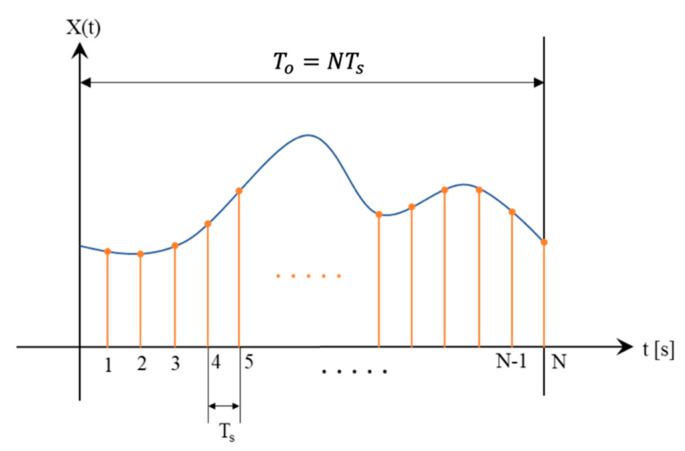
Sampling principle of an arbitrary signal.

According to the above definitions and to Figure 3, one can obtain Equation (6). Through simple mathematics it can be shown that $T_{s}$ is normalized in Equation (6) and it is the motivation to define a new variable $f_{n}$, the “normalized frequency”.

(6) $$\frac{f_{o}}{f_{s}}=\frac{1}{N}=f_{n}$$

Then, in light of Equations (5) and (6), it is possible to write:

(7) $$\omega_{k}=2\pi \frac{k}{N}=2\pi kf_{n}$$

Since $f_{o}$ and $f_{s}$ are known design parameters, according to Equation (6), $f_{n}$ is known as well. Moreover, *k* is ranging from 1 to *N*. Now Equation (6) can be rewritten in the general form as Equation (8), which $f_{o,k}$ is defined as the frequency corresponding to the sample number *k*.

(8) $$f_{o,k}=\frac{k}{N}f_{s}=kf_{n}f_{s}$$

From Equations (6) and (8), the relation between the sample number (*k*), time domain repetition frequency ($f_{o}$), and corresponding frequency to *k*th sample is summarized in:

(9) $$f_{o,k}=kf_{o}$$

which shows that the frequency corresponding to the *k*-th sample ($f_{o,k}$) is dependent of the sinc signal repetition frequency in time domain ($f_{o}$), that is the design parameter to be chosen. Moreover, Equation (9) indicates that the frequency resolution is exactly $f_{o}$.

### 4.2. Sinc Function Characteristic

The sinc function expressed in Equation (3) crosses the horizontal axis at each integer value of the amplitude variable. Considering the sinc function domain [−a,+a] such that $a\in {\mathbb{Z}}^{+}$, there will be 2a−1 extrema in that domain. One extremum appears at x=0 and (2a−2) extrema appear between each two consecutive integer points in domains [−a,−1] and [+1,+a]. The extrema points are maxima and minima alternatively. By convention, each extremum represents a lobe in the sinc function. Therefore, there is one “main lobe” and (a−1) “side lobe” at each side of the main lobe.

For a sinc signal with a numbers of side lobes and N samples, DFT amplitude outputs two rectangular windows with the width of a number of samples each and the approximated amplitude of N2a. An approximation of the DFT amplitude in terms of samples (n) for the considered sinc function can be generalized as:

(10) |FFT|={N2a1 ≤ n < a0a < n < N − aN2aN − a < n ≤ N

Considering the continuous sinc function defined in [−a,+a], negative numbers are considered, but in sampled sinc function, the samples range from 0 to *N*. For this reason, the DFT outputs two rectangular windows with the width of a for each window. In this case we only consider the first rectangle window from 0 to a, because the other rectangle corresponds to the negative frequencies in Fourier Transform analysis of the continuous sinc function and not the discrete one.

In this stage, according to Equation (9), we can specify the full frequency range of the designed sinc signal by considering k=a and selecting the proper value for $f_{o}$. Parameters $f_{o}$ and a are considered as design parameters and in this work a sinc function with a=50 and $f_{o}=50\;\mathrm{Hz}$ with *N* = 10,000 samples for each period is designed. Inserting the design parameters in Equation (9) leads to $f_{o,50}=2.5\;\mathrm{kHz}$ which is the power quality range. As for the selected a=50, it gives a sinc signal with 49 side lobes as shown in Figure 2.

### 4.3. Sinc Function Fourier Series Coefficients

In practice, the Fourier series (FS) coefficients for each harmonic are critical to be considered. FS coefficients represent the amplitude of each sine wave with different frequencies applied to the LPVT during the SR test. As the LPVT is designed to be supplied with sinusoidal waveforms, it is important to verify the LPVT operation under sinc function. To do this, the study of FS coefficients is necessary. In this work, the signal processing is based on DFT and to calculate the FS coefficients from DFT amplitude we need to divide it to the number of samples (N). Considering (10), the FS coefficients are approximated as 12a for all the harmonics. The 12a values are the amplitude of the sinusoidal waves which are applied to the LPVT during the SR test if the amplitude of sinc signal is one. To compute the ratio error and phase displacement in the test procedure in Section 6, the exact values are calculated and considered. Considering the design parameters a=50 and $f_{o}=50\;\mathrm{Hz}$, the FS coefficients are approximated as 12a=0.01 V, if the peak amplitude of sinc Signal is 1 V. In practice the sinc signal is amplified 16,000 times and by applying 16 kV sinc signal to the LPVT, the FS coefficient are about 160 V (0.01 × 16,000). In other words, while LPVT is supplied by the series of sinusoidal waveforms with the amplitude of 160 V, the LPVT is experiencing 16-kV peak value by injecting the 16 kV sinc signal, and this is one of the big advantages of SR test. Although the LPVTs are designed to be supplied with sinusoidal waveforms and sinc signal has a different voltage profile (in terms of RMS and mean values), in the results section it is shown that the LPVT behavior is the same as long as it experiences the same voltage stress related to the peak voltage.

## 5. Experimental Test Setup

**Figure 4:**
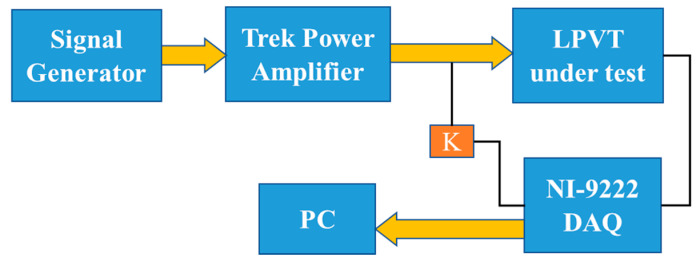
Experimental test setup scheme.

The designed sinc signal from last section is sampled and uploaded in a signal generator as an arbitrary waveform to be generated. The obtained signal is amplified by a power amplifier up to the rated peak voltage (16 kV). The amplified signal is applied to the LPVT under test as the primary voltage. The LPVT secondary voltage is acquired using a DAQ along with the primary voltage which is scaled using a resistive–capacitive reference voltage divider. Figure 4 represents the simplified scheme of the implemented test setup.

Its main components are:

- Agilent 33250A 80 MHz function/arbitrary signal generator used to generate the designed sinc signal;
- Trek high speed/high voltage power amplifier model 20/20C-HS to amplify the generated signal to 16-kV peak rated voltage. Its main characteristics are listed in Table 1 (see also [36]);
- Passive capacitive LPVT under test with $\frac{20}{\sqrt{3}}\;\mathrm{kV}/\frac{2.73}{\sqrt{3}}\;V$ rated voltage and 0.5 accuracy class;
- Resistive–capacitive reference voltage divider with $\frac{20}{\sqrt{3}}\;\mathrm{kV}/\frac{3.34}{\sqrt{3}}\;V$ rated voltage, ratio K and 0.1 accuracy class to measure the primary signal. It is used as a reference;
- NI 9222 data-acquisition board with ±10-V range and 500 kSa/s sampling frequency. Its accuracy features are: ±0.02% gain error and ±0.01% offset error.

## 6. Experimental Tests

The experimental tests are divided in three empirical test procedures including sinc-response (SR) test, single frequency (SF) test and distorted waveform (DW) test. SR test is the main test to extract the LPVT model by acquiring its transfer function. By using the transfer function, it is possible to predict the LPVT output for any inputs with frequency components up to 2.5 kHz. The prediction of the output is done by convolution between the acquired transfer function and the input signal in time domain.

The SF and DW tests are simply used for validation of the SR test and of the output prediction. The ratio error and phase displacement are then used as tools to validate the proposed approach.

### 6.1. SR Test Procedure

To perform the SR test, the designed sinc signal with a=50 is supplied to the Agilent 33,250A signal generator. The signal generator output is amplified using Trek Voltage amplifier with repetition frequency of $f_{o}=50$ Hz. The amplified signal (16 kV) is applied to the LPVT under test as the primary voltage.

Afterwards, the ratio error ε and the phase displacement ∆φ are computed as:

(11) $$\varepsilon =\frac{K\left|\vec{v_{2}}\right|-\left|\vec{v_{1}}\right|}{\left|\vec{v_{1}}\right|}100$$

(12) $$∆\varphi =∠\vec{v_{2}}-∠\vec{v_{1}}$$

in which $\vec{v_{2}}$ and $\vec{v_{1}}$ are the secondary and primary voltage phasors, respectively. $\vec{v_{2}}$ is measured directly from the LPVT output and $\vec{v_{1}}$ is measured using a resistive–capacitive reference voltage divider, which was characterized in a range of frequency that includes the power quality one.

Once both primary and secondary quantities are acquired, the DFT is applied and then ε and ∆φ are calculated for each frequency component from 50 Hz to 2.5 kHz with frequency step of $f_{o}=50$ Hz. Moreover, the frequency spectrum is saved for analytical operation to extract the transfer function and output prediction.

### 6.2. SF Test Procedure

The SF test is done using the same test setup as SR test, but with rated voltage pure sinusoidal waveforms with frequencies 50 Hz, 500 Hz and 1 kHz. For each frequency, ε and ∆φ are calculated to be compared with those computed after the estimation process. The SF test is limited to 1 kHz due to amplifier power limitation under the rated voltage; however, the LPVT linearity guarantees that if the method is effective up to 1 kHz, it will be effective even at 2.5 kHz.

### 6.3. DW Test Procedure

The distorted waveform test is another test for validation of the SR test and the results of the output prediction like SF test, except that in the DW test the fundamental harmonic at 50 Hz is superimposed to 3rd, 5th and 7th harmonics to simulate a distorted waveform in the power network. The amplitude of fundamental harmonic is set to the rated voltage, while the 3rd, 5th and 7th harmonics are set to the 10% of the rated voltage. Overall, 4 tests were performed: (i) three tests in which the 50 Hz component is applied when one of the three harmonics is superimposed; (ii) one test in which the three harmonics are superimposed at the same time to the 50 Hz. During these tests, ε and ∆φ are calculated for each frequency components using DFT analysis to be compared with the one from output prediction.

The DW test is included in the method validation procedure because LPVTs experience distorted voltages during their actual operation. Therefore, a method capable of estimating the LPVTs behavior even in distorted (hence more actual) conditions is more desirable than methods which simply works at rated conditions. As it is demonstrated in the next sections, the proposed approach is fully effective and applicable in all distorted conditions.

### 6.4. Output Prediction and Validation

The purpose is to predict the output of the LPVT using its sampled data transfer function in simulation. Finally, the predicted outputs are validated by the experimental test. Furthermore, the SR test is validated by SF and DW tests.

First, the transfer function is calculated using the SR test results. During the SR test, the DFT of the input sinc (IS) signal and sinc-response which are row vectors of complex numbers with the length of N = 10,000 samples, are saved in polar coordinate. Using:

(13) $$H\left(j\omega \right)=\frac{FFT\left(SR\right)}{FFT\left(IS\right)}=\frac{\left|FFT\left(SR\right)\right|}{\left|FFT\left(IS\right)\right|}\;\left(∠FFT\left(SR\right)-∠FFT\left(IS\right)\right)$$

the frequency domain transfer function (H(jω)) is calculated for the LPVT. Writing (11) in polar coordinates is useful to interpret Equation (11) as ε and ∆φ (|| and ∠ for the amplitude and phase, respectively). It is necessary to consider that the LPVT needs to have zero initial condition for the tests, and we are interested only in the steady state response of the LPVT. The calculated H(jω) is a vector of complex numbers with length *N* = 10,000 Sa.

By applying the inverse DFT, the time domain transfer function (h(t)) is found as a row vector of real numbers and length *N*. Afterwards, it is possible to apply the convolution

(14) $$ℱ\left\{h\left(t\right)\;\times \;x\left(t\right)\right\}\;=\;ℱ\dot{\dot{\left\{h\left(t\right)\right\}}}\;\times \;ℱ\left\{x\left(t\right)\right\}$$

to h(t) with any other LPVT input signal (x(t)) to predict the device output. The convolution theorem is the main motivation to use (11) to extract the transfer function and for this reason, the h(t) is referred as the model of LPVT to be validated by SR experimental test.

It is important to mention that the computed convolution is valid if and only if the generic input signal x(t) has a frequency content limited to 2.5 kHz. The reason is quite obvious when considering the synthetized sinc signal which has a bandwidth limited to 2.5 kHz.

#### Filtered Transfer Function

**Figure 5:**
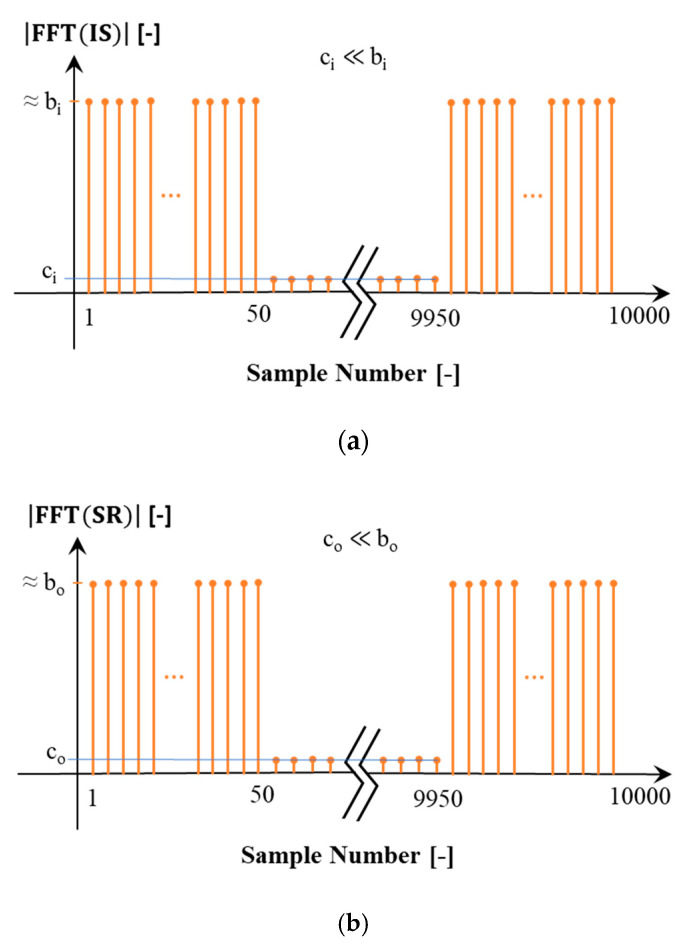

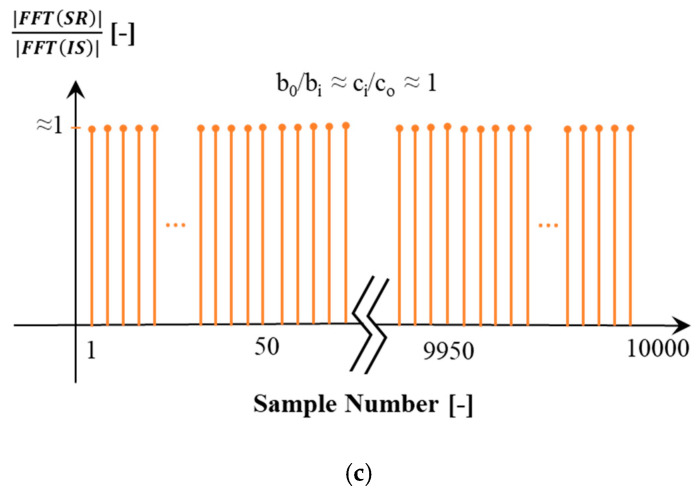
(**a**) DFT magnitude of the input sinc signal; (**b**) DFT magnitude of the sinc-response signal; (**c**) ratio between DFT magnitude of input sinc and sinc-response signal.

The purpose of this paragraph is to explain the removal of the noise frequency components higher than 2.5 kHz from the adopted signals. To shine some light on the topic, Figure 5 is considered as the amplitude of the DFT for both IS signal and SR signal (|FFT|). To use Equation (11) for calculating the frequency domain transfer function (H(jω)), the quotient between DFT of IS signal and SR signal showed in Figure 5 is computed. In Equation (11), |FFT(SR)| is the primary voltage measured by LPVT and |FFT(IS)| is the reference primary voltage measured by reference voltage divider. To calculate $\frac{\left|FFT\left(SR\right)\right|}{\left|FFT\left(IS\right)\right|}$, the two rectangular windows in Figure 5 are divided to each other. For frequencies higher than 2.5 kHz (sample numbers higher than 50) two small numbers c_i_ and c_o_ are divided to each other, while for the frequencies below 2.5 kHz (sample numbers from 1 to 50) high values of b_i_ and b_o_ (≈160 V) in rectangle width are divided to each other. The division of two small values (c_i_ and c_o_) turned out to be about 1 like the division of two high values in rectangle width is (b_o_/b_i_ ≈ c_o_/c_i_ ≈1).

One can interpret this flat profile in all the frequency rang as a normalized impulse-response related to the ratio error of the LPVT in all frequencies. The approach to remove the effect of the ratio error in high frequencies is to consider zero ratio error (fixing $\frac{\left|FFT\left(SR\right)\right|}{\left|FFT\left(IS\right)\right|}=1$) and zero phase displacement (∠FFT(SR) − ∠FFT(IS) = 0) for frequencies higher than 2.5 kHz. The two filtered and unfiltered versions of h(t) represent the same values for frequencies below 2.5 kHz. In what follows only the filtered version is used.

## 7. Results

### 7.1. SR Experimental Test Results

**Figure 6:**
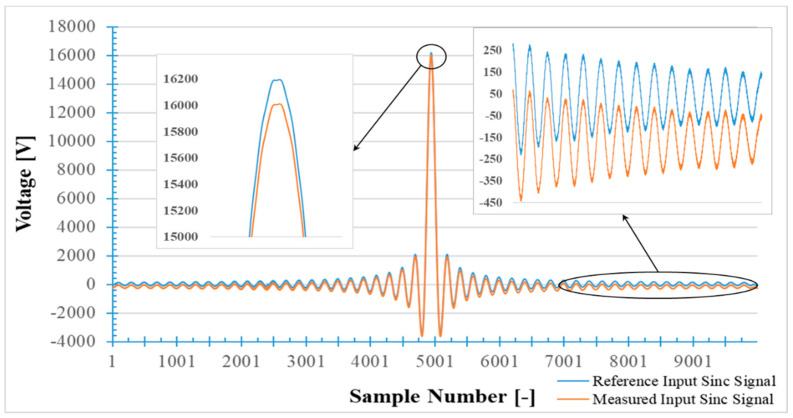
Reference input sinc signal applied to the LPVT under test, measured by reference resistive–capacitive voltage divider and the input sinc signal measured by the LPVT under test (LPVT sinc-response transferred to the primary side).

The primary IS signal was applied to the LPVT under test as the primary signal and the output signal (SR signal) multiplied by the rated transformation ratio was the measured primary voltage. Figure 6 shows both IS and SR signals in time domain. Using the DFT of both IS and SR signals, it was possible to calculate ε and ∆φ for all frequency components from 50 Hz to 2.5 kHz with steps of 50 Hz. For the sake of clarity and of comparison in what follows, for some frequencies ε and ∆φ are listed in Table 2. The values in the table are the mean of 500 computations; furthermore, the values are given with their standard deviation of the mean $\sigma_{m}$. From the table it is possible to conclude that the LPVT under test was properly operating within the limits of its accuracy class (0.5). It is worth clarifying that the DC offset observable in Figure 6 is mainly due to the instrumentation adopted for acquiring the primary and the secondary quantities. However, it was not significant in the experimental resulted as it was confirmed by the resulted in Table 2.

### 7.2. Transfer Functions

**Figure 7:**
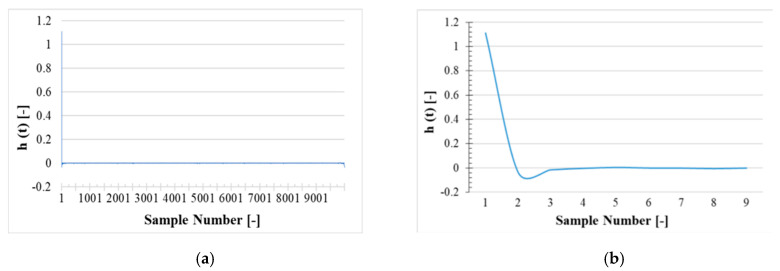

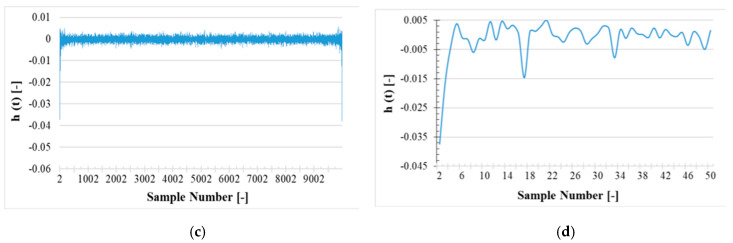
Time domain unfiltered version of The LPVT sampled data transfer function. (**a**) all 10,000 samples; (**b**) first 9 samples; (**c**) only the first sample removed; (**d**) samples from the 2nd to the 50th.

**Figure 8:**
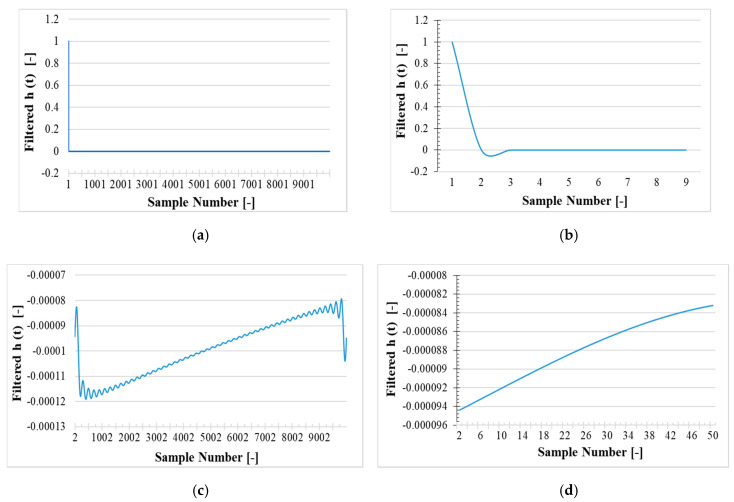
Time domain filtered version of the LPVT sampled data transfer function. (**a**) all 10,000 samples; (**b**) first 9 samples; (**c**) only the first sample removed; (**d**) samples from the 2nd to the 50th.

The time domain transfer function (h(t)) calculated by performing the inverse DFT on H(jω) in Equation (11), is divided in two filtered and unfiltered versions. The results of the filtered version are graphed in Figure 7, while those of the unfiltered ones are plotted in Figure 8. In both cases and figures, 4 zoomed parts of the graphs can be distinguished.

This is mainly due to (i) the high number of samples and (ii) to the sharp variations of the transfer function in the time domain. In both Figure 7 and Figure 8, the fast reduction of h(t) magnitude from first sample to the second sample is clear. This is a typical impulse-response for a linear-time invariant (LTI) system. The reason for the fast decreasing of h(t) magnitude is the slightly frequency dependence feature of the LPVT under test.

Let us consider a frequency independent system impulse-response which is another impulse signal in the system output. In this case the system transfer function amplitude in frequency domain (|H(jω)|) will be a fixed gain and in the time domain, h(t) represents an impulse. This is behavior is clearly seen in Figure 7b and Figure 8b.

Discarding the fast reduction in h(t) magnitude, it can be seen in Figure 7c and Figure 8c that the variation from second sample to the end is much smaller than the fast drop of the h(t) magnitude from the first to the second sample. To magnify the variation of the h(t) magnitude from the 2nd sample to the 50th sample, Figure 7d and Figure 8d are presented.

Figure 8 features a smooth profile due to the filtering action on ε and ∆φ for samples number higher than 50 (corresponding to frequencies higher than 2.5 kHz). The filtering action refers to the ε and ∆φ set to zero for frequencies higher than 2.5 kHz. Moreover, the filtering action in Figure 8 has reduced the variation of h(t) magnitude compared to Figure 7.

### 7.3. SF Experimental Test Results

Table 3 shows ε and ∆φ results for SF test under 50 Hz, 500 Hz and 1 kHz at rated voltage ($20/\sqrt{3}$ kV). The table contains the mean value and standard deviation of 500 ε and ∆φ measurements. From the table, it is evident that the obtained ε and ∆φ values confirms those listed in Table 2, which are computed by using the SR test (variations below 10 %). In other words, this first test confirms the applicability of the simple SR test.

### 7.4. DW Experimental Test Results

The DW test is performed using the same test setup and a distorted waveform as primary voltage. This set of tests contains 4 different tests with different composition of the harmonics superposed to the fundamental harmonic (50 Hz). ε and ∆φ results for each frequency component are reported in Table 4.

In all harmonic compositions, the amplitude of the fundamental harmonic is set to the rated value and the amplitude of the 3rd, 5th and 7th harmonics is set to 10% of the rated voltage. In all Cases 500 ε and ∆φ are computed and mean values are reported in Table 4 along with the combined uncertainty. The results show that the LPVT under distorted condition still holds the accuracy class of 0.5 and it validates the results achieved from SR test reported in Table 2 for the same frequency composition. Of course, such a validation is only preliminary and secondary; as a matter of fact, the DW test is aimed at validating the estimation approach presented in the next subsection.

### 7.5. Output Prediction and Validation

The output prediction is simulated in software environment by synthesizing the input signals, adopted for the SF and DW experimental tests and then convolute them with the calculated transfer function in time domain (h(t)). The purpose is to use the LPVT model Transfer function (h(t)) in software environment to predict the LPVT output signal, validating it by computing ε and ∆φ and comparing it with those obtained from the experimental results. The estimated ratio error and phase displacement are denoted with $\hat{\varepsilon }$ and $\hat{\Delta \varphi }$.

#### 7.5.1. SF Test Prediction

**Figure 9:**
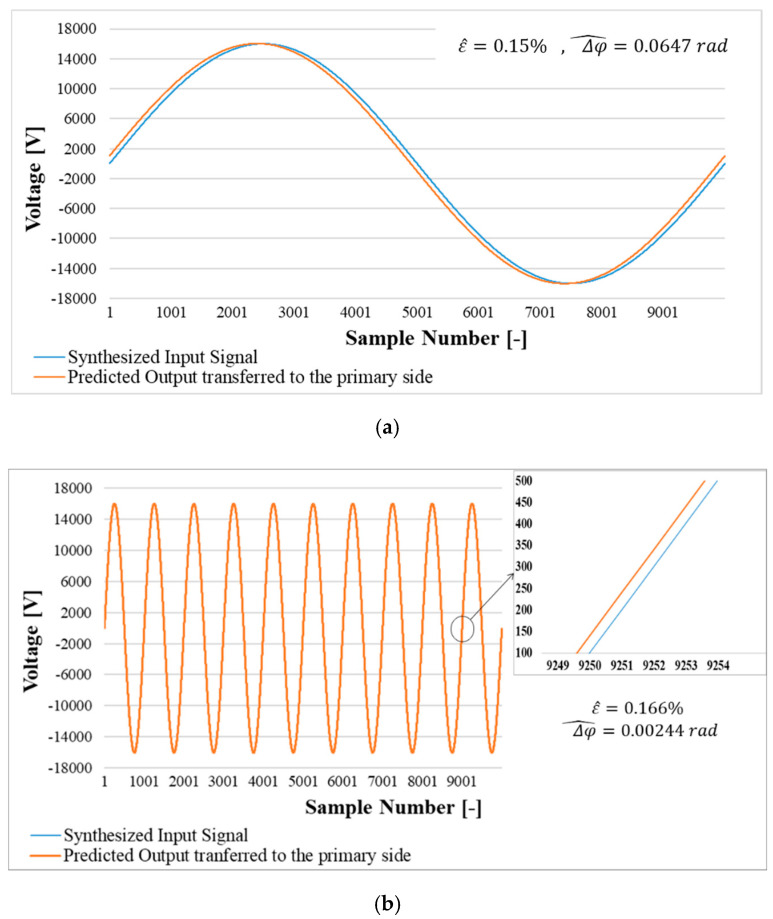

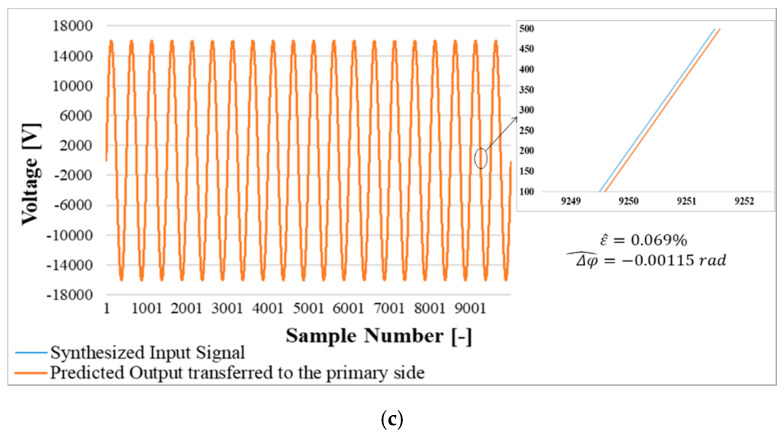
Synthesized sinusoidal input signal and the measured signal prediction by simulation. (**a**) prediction for 50 Hz; (**b**) prediction for 500 Hz; (**c**) prediction for 1 kHz.

For each of the 3 frequencies described in Section 6.2, a waveform was synthetized and convoluted with h(t). Then, $\hat{\varepsilon }$ and $\hat{\Delta \varphi }$ were computed in all cases. Figure 9 shows both synthetized input signals and predicted outputs along with the estimated $\hat{\varepsilon }$ and $\hat{\Delta \varphi }$.

For the sake of comparison, Table 5 reports both the $\hat{\varepsilon }$ and $\hat{\Delta \varphi }$ estimated in this section and the ε and ∆φ obtained during the experimental SR tests. From table it can be appreciated the efficiency and validity of the estimation approach for both the ratio error and the phase displacement.

#### 7.5.2. DW Test Prediction

**Figure 10:**
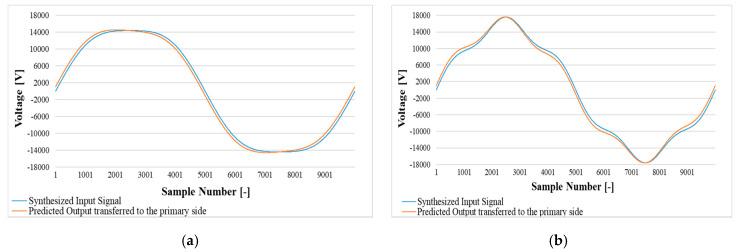

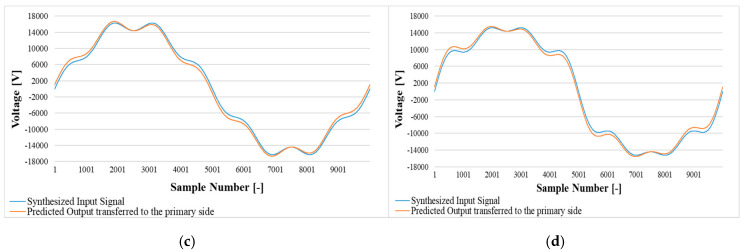
Synthesized distorted input signal and the measured signal prediction by simulation. (**a**) prediction for 1st + 3rd harmonics; (**b**) prediction for 1st + 5th harmonics; (**c**) prediction for 1st + 7th harmonics; (**d**) prediction for 1st + 3rd + 5th + 7th harmonics.

For the distorted waveforms introduced in the DW tests, the estimation process described in Section 7.5.1 was applied too, obtaining another set of estimated $\hat{\varepsilon }$ and $\hat{\Delta \varphi }$. Figure 10 shows, for each synthetized distorted signal, the primary voltage and the estimated secondary one, scaled to the primary side. Starting from those waveforms, $\hat{\varepsilon }$ and $\hat{\Delta \varphi }$ were computed (estimated) for each case and listed in Table 6. Table must be compared with Table 4 (and also with Table 2) to appreciate the obtained estimates. Note that the estimated and experimental values are almost identical and confirm, even in the case of distorted waveforms, the efficiency and applicability of the estimation approach.

As a final comment, it is worth summarizing the main achievements of this work:

- The SR approach was validated with typical tests methods like SF and DW. By means of ε and ∆φ it was demonstrated the high accuracy and validity of the proposed SR test;
- The SR test combined with the mathematical convolution were implemented to estimate the output of the LPVT under test plus its two main accuracy indices: $\hat{\varepsilon }$ and $\hat{\Delta \varphi }$;
- All experimental tests were compared with the associated estimated ones demonstrating that, in all cases, the proposed approach provides very accurate results.

## 8. Conclusions

The study—after presenting related theoretical aspects—describes the experimental setup and tests performed on a low-power voltage transformer. The sinc-response is used to obtain a transfer function, hence a model of the device, which allows the estimation of the transformer output in whatever operating condition. Such an approach was validated comparing experimental measurements and estimations at: (i) rated conditions, (ii) frequencies different from the rated, (iii) distorted conditions. What was demonstrated with the results is that the developed approach is effective and applicable in all above mentioned operating conditions. Therefore, what proposed here can become a useful and powerful tool to improve the characterization of voltage transformers and to be implemented inside simulation software used by distributed system operators, utilities and academic people.

## Figures and Tables

**Table 1:** Trek power amplifier features.

Output Voltage Range	0 to 20–kV DC or AC Peak	DC Voltage Gain	20,000 V/1 V
Input voltage range	0 to 10–V DC or AC peak	Drift with time	<50 ppm/h
DC voltage gain accuracy	<0.1% of full scale	Slew rate	800 V/µs
Drift with temperature	<100 ppm/°C	Signal bandwidth	DC to 5.2 kHz

**Table 2:** Experimental SR test results in terms of ε and ∆φ.

*f* (Hz)	*ε* (%)		Δ*φ* (rad)	
	Mean Value	$\sigma_{m}$	Mean Value	$\sigma_{m}$
50	0.015	0.02	0.0647	0.0001
150 (3rd harmonic)	0.28	0.01	0.0187	0.0001
250 (5th harmonic)	0.246	0.009	0.00947	0.00009
350 (7th harmonic)	0.212	0.008	0.00548	0.00008
500	0.166	0.008	0.00244	0.00008
1000	0.069	0.007	−0.00115	0.00007

**Table 3:** Experimental SF test results.

*f* (Hz)	*ε* (%)		Δ*φ* (rad)	
	Mean Value	$\sigma_{m}$	Mean Value	$\sigma_{m}$
50	0.1435	0.0002	0.064691	0.000002
500	0.1638	0.0002	0.002444	0.000002
1000	0.0761	0.0002	−0.001190	0.000002

**Table 4:** Experimental DW test results.

Test	Component	*ε* (%)		Δ*φ* (rad)	
		Mean Value	$\sigma_{m}\;$	Mean Value	$\sigma_{m}\;$
50 Hz + 3rd	50 Hz	0.1475	0.0002	0.064736	0.000001
	150 Hz	0.3103	0.0008	0.018602	0.000008
50 Hz + 5th	50 Hz	0.1396	0.0001	0.064723	0.000001
	250 Hz	0.2692	0.0007	0.009485	0.000007
50 Hz + 7th	50 Hz	0.1406	0.0002	0.064721	0.000001
	350 Hz	0.2327	0.0006	0.005461	0.000006
50 Hz + all	50 Hz	0.1409	0.0001	0.064719	0.000001
	150 Hz	0.3083	0.0008	0.01857	0.00001
	250 Hz	0.2691	0.0008	0.009433	0.000007
	350 Hz	0.2302	0.0007	0.005462	0.000007

**Table 5:** SF Prediction results and comparison with SR experimental test results.

*f* (Hz)	SR Experimental Test Results		SF Prediction Results by Simulation	
	*ε* (%)	Δ*φ* (rad)	$\hat{\varepsilon }$ (%)	$\hat{\Delta \varphi }$ (rad)
50	0.15	0.0647	0.15	0.0647
500	0.166	0.00244	0.166	0.00244
1000	0.069	−0.00115	0.069	−0.00115

**Table 6:** DW prediction results.

Test	Component	$\hat{\varepsilon }$	$\hat{\Delta \varphi }\;(\mathrm{rad})$
50 Hz + 3rd	50 Hz	0.15	0.0647
	150 Hz	0.28	0.0187
50 Hz + 5th	50 Hz	0.15	0.0647
	250 Hz	0.246	0.00947
50 Hz + 7th	50 Hz	0.15	0.0647
	350 Hz	0.212	0.00548
50 Hz + all	50 Hz	0.15	0.0647
	150 Hz	0.28	0.0187
	250 Hz	0.246	0.00947
	350 Hz	0.212	0.00548
